# Minnesota State Medical Society

**Published:** 1875-02-15

**Authors:** 


### MINNESOTA STATE MEDICAL SOCIETY.

THE State Medical Society of Minnesota assembled in regular session at St. Paul, February 2d, 1875, the President, Dr. N. B. Hill, of Minneapolis, in the chair, and Dr. Charles E. Smith, of St. Paul, acting as Secretary.

During the recess between the morning and afternoon sessions, the President, Dr. Hill, was seized with an apoplectic fit, which has since proved fatal.

Upon re-assembling in the afternoon it was evident that the sudden illness of the President had caused a feeling of profound regret among his associates of the profession, and to fill the unexpected vacancy caused thereby, Dr. A. B. Stuart, of Winona, Vice-President, was called to the chair and presided during the afternoon, after announcing the sad event which made the course necessary.

At the evening session the chairman of the committee on diseases of the nervous system, Dr. H. C. Hand, reported instances of successful treatment by hypodermic injection of morphine, which report was adopted and referred to the publication committee.

Dr. Hoyt, of Hudson, presented a case of ovariotomy of a remarkable character, and another of a thyroid tumor or goitre removed from the neck of a lady. The doctor's trepidation was curtly expressed in the words "That he was scared, for she bled like blazes," but finally got well.

At the second day's session, the President's address was read by Dr. C. N. Hewitt, who explained the circumstances under which he had obtained it from the esteemed gentleman who was at the moment lying in such a critical condition at the residence of Dr. J. H. Murphy.

Dr. Hewitt proceeded with the reading of the address, which had come into his possession under such painful circumstances, and which was listened to with marked attention by President Hill's professional brethren assembled on this occasion. The address covered a wide range of subjects of special value to members of the profession, and at the conclusion of the reading it was referred to a special committee to be appointed for the consideration of the several subjects embraced therein—the committee being appointed by the chair, and consisting of Drs. Boardman, Evans, Staples, Hewitt and Milligan.

The following officers were elected for the ensuing year: President, J. H. Stewart, of St. Paul; First Vice-President, Dr. Otis Ayer, of La Sueur; Second Vice-President, Dr. A. C. Wedge, of Albert Lea; Third Vice-President, Dr. D. W. Hand, of St. Paul; Treasurer, Dr. S. B. Sheardown, of Winona; Secretary, Dr. Charles E. Smith, of St. Paul; Cor. Secretary, Dr. A.W. Stinchfield, of Eyota, Olmsted county.

Dr. Stone read a paper exhibiting the effect of certain diseases upon the eye, which had been prepared by Dr. Ole Bull, of Minneapolis. The paper was ordered published, and the thanks of the Society were returned to Dr. Bull for his valuable and interesting contribution.

The formal report of the case of ovariotomy which had been referred in the first day's proceedings, as having been under the treatment of Dr. Otis Hoyt, of Hudson, was read by the Secretary, and received the undivided attention of the profession—the case being one of remarkable character, and the surgical operation for removal comparatively new and entirely successful. The reading of the report was concluded in the midst of applause. It was ordered published, and the thanks of the Society were returned to Dr. Hoyt for his interesting contribution to the scientific literature of the Society.

A report of the removal of a tumor by Dr. A. B. Stuart, was next read by the Secretary, and referred to the publication committee.

On motion of Dr. Hand, the Society adjourned till the next annual meeting.

